# Validation of the Japanese Big Five Scale Short Form in a University Student Sample

**DOI:** 10.3389/fpsyg.2022.862646

**Published:** 2022-06-23

**Authors:** Rie Toyomoto, Masatsugu Sakata, Kazufumi Yoshida, Yan Luo, Yukako Nakagami, Taku Iwami, Shuntaro Aoki, Tomonari Irie, Yuji Sakano, Hidemichi Suga, Michihisa Sumi, Hiroshi Ichikawa, Takafumi Watanabe, Aran Tajika, Teruhisa Uwatoko, Ethan Sahker, Toshi A. Furukawa

**Affiliations:** ^1^Department of Health Promotion and Human Behavior, School of Public Health, Kyoto University Graduate School of Medicine, Kyoto, Japan; ^2^Agency for Health, Safety and Environment, Kyoto University Health Service, Kyoto, Japan; ^3^Center for Medical Education and Career Development, Fukushima Medical University, Fukushima, Japan; ^4^Department of Psychology and Counseling, School of Education and Culture, Hokusho University, Ebetsu, Japan; ^5^Department of Clinical Psychology, Goryokai Medical Corporation, Sapporo, Japan; ^6^Department of Social Welfare, Ryukoku University Junior College, Kyoto, Japan; ^7^Ritsumeikan Medical Service Center, Kyoto, Japan; ^8^Department of Medical Life Systems, Faculty of Life and Medical Sciences, Doshisha University, Kyoto, Japan; ^9^Department of Psychiatry and Cognitive-Behavioral Medicine, Nagoya City University Graduate School of Medical Science, Nagoya, Japan; ^10^Department of Psychiatry, Kyoto University Hospital, Kyoto, Japan; ^11^Medical Education Center, Kyoto University Graduate School of Medicine, Kyoto, Japan

**Keywords:** personality, psychometric properties, five-factor model, adolescence, brief measures

## Abstract

The Japanese Big Five Scale Short Form (JBFS-SF), a 29-item self-report scale, has recently been used to measure the Big Five personality traits. However, the scale lacks psychometric validation. This study examined the validity and reliability of the JBFS-SF with data collected from 1,626 Japanese university students participating in a randomized controlled clinical trial. Structural validity was tested with exploratory and confirmatory factor analysis and measurement invariance tests were conducted across sex. Internal consistency was evaluated with McDonald’s omega. Additionally, construct validity was estimated across factors using the PHQ-9, GAD-7, AQ-J-10, and SSQ. EFA results showed that the JBFS-SF can be classified according to the expected five-factor structure, while three items had small loadings. Therefore, we dropped these three items and tested the reliability and validity of the 26-item version. CFA results found that a 26-item JBFS-FS has adequate structural validity (GFI = 0.907, AGFI = 0.886, CFI = 0.907, and RMSEA = 0.057). The omega of each factor was 0.74–0.85. Each JBFS-SF factor was specifically correlated with the PHQ-9, GAD-7, and SSQ. This research has shown that the JBFS-SF can be a clinically useful measure for assessing personality characteristics.

## Introduction

The Big Five Model (Goldberg, 1993) and the Five-Factor Model (McCrae and Costa, 1987) are two of the foremost theories for classifying personality traits. The models consist of five basic dimensions: Openness to experience (e.g., tendency to be creative, intellectually curious, and emotionally open), Conscientiousness (e.g., self-disciplined, organized, and reliable), Extraversion (e.g., energetic, enthusiastic, and socially outgoing), Agreeableness (e.g., thoughtful, kind, and modest), and Neuroticism (e.g., emotionally unstable and experiencing negative emotions). It has been widely used and had a significant impact on the research of individual personality traits. Previous studies have shown that these personality traits are associated with various individual health-related outcomes including wellbeing, stress responses, depression, and anxiety (Soto, 2019). Personality traits are also reported to be related with a broad range of interpersonal and social variables, such as autistic traits (Lodi-Smith et al., 2019) and perceived social support (Barańczuk, 2019). Furthermore, personality traits can influence treatment adherence and outcomes (Maj et al., 2020).

Based on the Big Five Model or Five-Factor Model, various scales, such as the 240-item Revised NEO Personality Inventory (NEO-PI-R; Costa and McCrae, 1992), the 60-item NEO Five-Factor Inventory (NEO-FFI; Costa and McCrae, 1992), and the 44-item Big Five Inventory (BFI-44; John and Srivastava, 1999), have been developed. However, these scales have many items, which are time-consuming and may cause stress and exhaustion leading to incompleteness or inaccuracy (Burisch, 1984). In recent years, shorter versions have been developed, such as the 10-item Big Five Inventory (BFI-10; Rammstedt and John, 2007) and the 20-item Mini-IPIP (Donnellan et al., 2006). Short versions can reduce the burden on the respondent, making it easier to use in conjunction with other measures, and can also lead to more accurate responses (Burisch, 1984). However, such advantages may be accompanied by a trade-off with psychometric weaknesses. Additionally, the short-form instruments listed above lack the cross-cultural support that the full-scale versions previously achieved.

In Japan, there are two types of scales: those translated from other countries’ scales [e.g., the 240-item Japanese NEO-PI-R (Shimonaka et al., 1998)] and those adapted to Japanese culture [e.g., the 150-item Five-Factor Personality Questionnaire (FFPQ; FFPQ Kenkyukai, 2002)] and the 60-item Japanese Big Five Scale (Wada, 1996).

The Japanese Big Five Scale (JBFS) was developed using the Adjective Check List (300 items; Gough and Heilbrun, 1983) and consists of 12 items for each factor. The JBFS has the advantage of being in adjective form, which makes the structure more stable than in sentence form (Gough and Heilbrun, 1983). To reduce the burden on respondents, the JBFS was shortened to 29 items using Item Response Theory (IRT) and reported to be highly correlated with the original JBFS (0.93–0.95; Namikawa et al., 2012). The Big Five Scale Short Form (JBFS-SF) has recently been used for the general population (Suzuki et al., 2015; Yoshino et al., 2021) and university students (Fukase et al., 2021; Hatabu et al., 2021). However, the reliability and validity of the JBFS-SF remain questionable because a part of the original scale (60 items, 7-point scale) was substituted for the validation of the short form, and the JBFS-SF itself (29 items, 5-point scale) has not been used. In addition, correlations with the JBSF-SF and NEO-FFI were low for some subscales (Openness 0.21, Agreeableness 0.46, Conscientiousness 0.52, Neuroticism 0.70, Extraversion 0.74; Namikawa et al., 2012).

In the present study, we fill the gap in the literature by investigating the psychometric properties of the JBSF-SF. We have included this JBFS-SF in a battery of psychological tests used for a clinical trial with university students and hereby aim to examine its reliability and validity.

## Materials and Methods

### Participants and Procedures

This study is based on data from the Healthy Campus Trial (HCT), which was a fully factorial randomized controlled trial to optimize smartphone cognitive behavioral therapy (CBT) among healthy university students. The HCT began in September 2018 and the final participant was enrolled in June 2021. Participants were recruited *via* posters, flyers, websites, and social networking services. Participants were included in the HCT if they were 18- to 39-year-old junior college, undergraduate, or graduate students from five universities in Japan, understood written Japanese, had a score of 0 to 14 on the PHQ-9, and were not receiving any mental health treatment at enrolment. Those scoring 15 or above were not eligible because such high scores indicate a high likelihood of clinical depression (Kroenke et al., 2001). Participants attended a group orientation meeting and provided written informed consent. After March 2020, due to the COVID-19 pandemic, we changed from face-to-face to online orientation meetings. Details of the trial are described in the protocol (Uwatoko et al., 2018). This trial is registered with UMIN-CTR Clinical Trial, CTR-000031307. Ethical approval was obtained from the Kyoto University School of Medicine.

We divided our sample into two groups. Sample 1 included 845 university students who participated in the HCT between September 2018 and May 2020 when half of the planned sample size was reached. Sample 2 included 781 students between June 2020 and June 2021. Sample 1 was used for the primary analysis and Sample 2 for a confirmatory factor analysis of the shortened 26-item JBFS.

### Measurement

All data were collected online *via* the trial website and smartphone application. We used a system that would not allow them to proceed unless they completed the responses, thus there were no missing values except for the Social Support Questionnaire.

#### The Japanese Big Five Scale Short Form

The Japanese Big Five Scale Short Form (JBFS-SF; Namikawa et al., 2012) is a brief version of the original 60-item JBFS (Wada, 1996) for assessing personality traits. The scale consists of 29 items on five personality trait factors: Openness to experience, Conscientiousness, Extraversion, Agreeableness, and Neuroticism. The items are composed of single adjectives in the adjective checklist form (Gough and Heilbrun, 1983), which avoids ambiguity and multiple meanings. Though the original JBFS-SF is a 5-point Likert scale from 1 (untrue of me) and 5 (true of me), we changed it to a 4-point scale for the following reasons: (1) to avoid a central tendency of odd-numbered Likert scales, and (2) to reduce the burden on respondents. A higher average score indicates a higher level of the self-reported trait. It has been reported that there is a high correlation (rs = 0.92–0.95) between each factor in the original and short versions (Namikawa et al., 2012).

#### The Patient Health Questionnaire-9

The Patient Health Questionnaire-9 (PHQ-9; Kroenke et al., 2001) is a self-reported questionnaire for screening and measuring severity of depression, based on the Diagnostic and Statistical Manual of Mental Disorders, Fourth Edition (DSM-IV) for major depressive disorder. It is a 9-item scale, each rated from 0 (not at all) to 3 (nearly every day). The reliability and validity of the original scale and the Japanese version have been demonstrated (Kroenke et al., 2001; Muramatsu et al., 2018). Previous studies showed that high neuroticism, which refers to the persistent tendency to experience negative emotions, is strongly associated with depressive symptoms, and we expected a positive correlation between PHQ-9 and Neuroticism (Kotov et al., 2010; Hakulinen et al., 2015; Soto, 2019).

#### The Generalized Anxiety Disorder Screener-7

The Generalized Anxiety Disorder Screener-7 (GAD-7; Spitzer et al., 2006) is a self-reported questionnaire for measuring anxiety. The GAD-7 comprises 7 items rated on a 3-point scale, ranging from 0 (not at all) to 3 (nearly every day). The validity and reliability of the original scale (Spitzer et al., 2006) and the Japanese version (Doi et al., 2018) have been established. Neuroticism has a robust association with anxiety, hence we assumed that the GAD-7 and Neuroticism would be positively correlated (Kotov et al., 2010; Watson and Naragon-Gainey, 2014; Soto, 2019).

#### The Autism Spectrum Quotient Japanese Version

The Autism Spectrum Quotient Japanese Version (AQ-J-10) is a short version of the Autism Spectrum Quotient measuring autistic traits (Baron-Cohen et al., 2001). The reliability and validity of the Japanese original version and its shortened version have been verified (Kurita et al., 2005). A recent meta-analysis indicated ASD characteristics were negatively associated with all Big Five personality traits, especially Extraversion (Lodi-Smith et al., 2019). Therefore, we expected a negative relationship between Extraversion and the AQ-J-10.

#### The Social Support Questionnaire

The Social Support Questionnaire (SSQ) has six items measuring the quality and quantity of social support (Sarason et al., 1987). The reliability and validity of the original scale and the Japanese version have been demonstrated (Sarason et al., 1987; Furukawa et al., 1999). A previous meta-analysis shows that Extraversion was positively associated with perceived support received, and we expected that the SSQ and Extraversion would be positively correlated (Barańczuk, 2019). We failed to include the second and fourth items from the original version among the first 917 participants due to a clerical error: we corrected the questionnaire after the 917th student. Therefore, when calculating correlation coefficients with the JBFS-SF in Sample 1, we substituted the subscale scores of the original six items by the average of the four items.

### Statistical Analysis

#### Structural Validity

In Sample 1, we assessed the structural validity of the JBFS-SF by conducting an exploratory factor analysis (EFA) using the psych package (Version 2.0.12) in R (Version 4.0.3; Revelle, 2017). EFA was performed with the principal factor method and Varimax rotation and the number of factors extracted was determined by a Scree test and the Kaiser criterion. We set the threshold for meaningful factor loadings at 0.4 for interpretative purposes (Stevens, 2012). If items were found to have small loadings of less than 0.4, we would exclude them and examine the validity of the scale composed of the remaining items.

In Sample 2, we conducted confirmatory factor analyses (CFA) to examine the model fit of the original and revised versions, first for the original version with all the items, and second for the revised version excluding the items which had loadings less than 0.4. We introduced covariant relationships where appropriate to improve the model fit. We used the lavaan package (version 0.6-8) in R to check the goodness-of-fit of the factor structure, goodness-of-fit index (GFI), adjusted goodness-of-fit index (AGFI), comparative fit index (CFI), and root mean square error of approximation (RMSEA; Rosseel, 2012). It is recommended that the criteria for a good model fit are GFI ≥ 0.95, AGFI ≥0.90, CFI ≥ 0.97, and RMSEA ≤0.05, and an acceptable model fit is GFI ≥ 0.90, AGFI ≥0.85, CFI ≥ 0.95, and RMSEA ≤0.08 (Schermelleh-Engel et al., 2003). We used AMOS 27 (IBM SPSS Statistics, Chicago, Illinois, United States) for graphics. Additionally, we conducted measurement invariance tests across sex using a series of multi-group CFAs, considering different tendencies of personality traits by sex (Schmitt et al., 2017). We compared the fit between four models (configural invariance, metric invariance, scalar invariance, and error variance invariance) using the likelihood ratio test and examined the differences in the comparative fit index (ΔCFI) and the Tucker–Lewis index (ΔTLI), which is not influenced by sample size. Non-significant *p*-values (*p* ≥ 0.05) in the chi-square difference test indicated that measurement invariance was acceptable (Bontempo and Hofer, 2007). ΔCFIs ≤ −0.01 and ΔTLIs ≤ −0.01 were interpreted as evidence of non-invariance (Cheung and Rensvold, 2002).

#### Internal Consistency Reliability

In Sample 1, we examined the internal consistency of the JBFS-SF by calculating McDonald’s omega (McDonald, 1999). We calculated omega rather than Cronbach’s alpha because many methodological studies suggest that Cronbach’s alpha has problems arising from unrealistic assumptions, such as one-dimensionality and homogeneity of scales (McNeish, 2018).

#### Construct Validity

In Sample 1, we calculated Pearson’s correlation coefficients between each factor of the JBFS-SF with the PHQ-9, GAD-7, AQ-J-10, and SSQ to examine our expectations mentioned in the “Measurement” section.

## Results

### Sample Characteristics

Table 1 summarizes the demographic and baseline characteristics of both samples. In Sample 1 (*N* = 845), the mean age (SD) was 22.0 (3.04), slightly more than half were women (53.4%), and most were in bachelor’s (70.1%) or master’s programs (21.7%). In Sample 2 (*N* = 781), the mean age (SD) was 21.0 (2.84), with more women (61.6%) and more students in bachelor’s programs (83.5%) than in Sample 1.

**Table 1:** Demographic and baseline characteristics.

	Sample 1 (*n* = 845)	Sample 2 (*n* = 781)
Age, mean (SD, range)	22.0 (3.04, 18–39)	21.0 (2.84, 18–39)
Sex		
Female	451 (53.4%)	481 (61.6%)
Male	394 (46.6%)	300 (38.4%)
Educational program		
Bachelor	592 (70.1%)	652 (83.5%)
Master	184 (21.7%)	109 (14.0%)
Doctoral	66 (7.8%)	19 (2.4%)
Junior college	3 (0.4%)	1 (0.1%)
PHQ-9, mean (SD)	6.36 (3.38)	6.41 (3.44)
GAD-7, mean (SD)	5.32 (3.24)	5.66 (3.48)

*PHQ-9; Patient Health Questionnaire-9, GAD-7; Generalized Anxiety Disorder Screener-7.*

### Validation of the 29-Item JBFS-SF

#### Structural Validity

**Figure 1:**
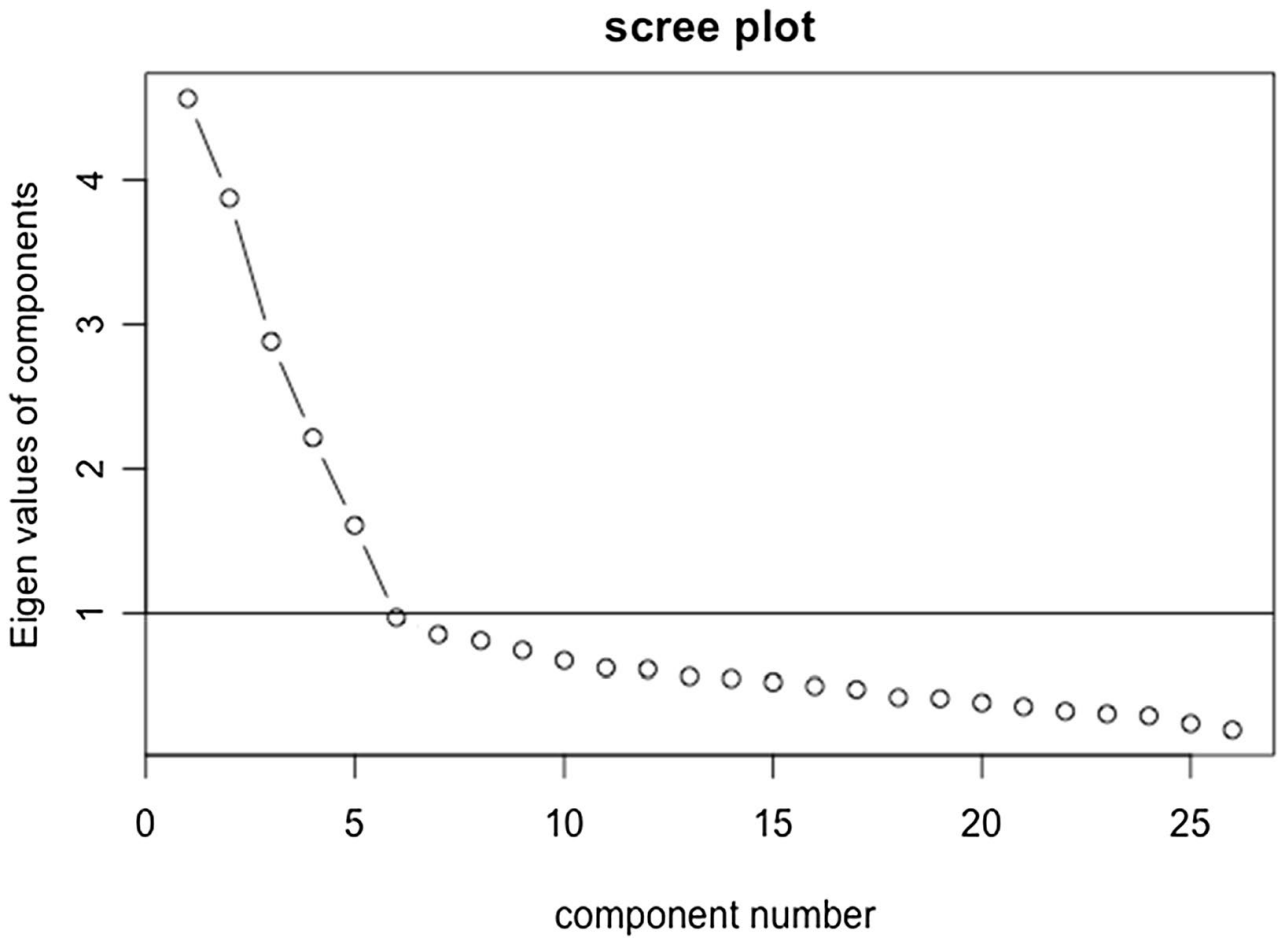
Scree plot of exploratory factor analysis.

As shown in Table 2, the EFA in Sample 1 extracted five factors for the JBFS-SF and the factor loadings for items of each factor were highest among the items as per the original scale: 0.359–0.760 for Conscientiousness, 0.613–0.831 for Extraversion, 0.358–0.761 for Agreeableness, 0.390–0.675 for Openness to experience, and 0.500–0.798 for Neuroticism. Item 25 of the JBFS-SF had an effect across the two factors of Conscientiousness and Agreeableness, and three items (items 19: sharp-witted, 25: self-centered, and 28: kind) had loadings of less than 0.4. Figure 1 shows the Scree plot. The sixth component has an Eigenvalue smaller than 1.0 and the plot flattens thereafter. We therefore decided to set the number of factors to extract at five (Kaiser, 1960). The results of CFA in Sample 2 assuming a covariant relationship between some error variables showed that the GFI = 0.856, AGFI = 0.827, CFI = 0.839, and RMSEA = 0.071.

**Table 2:** Factor loadings of the 29-item Big Five Scale Items with Varimax Rotation.

Item	Big Five Scale Subscale	Factor 1	Factor 2	Factor 3	Factor 4	Factor 5	Communality
2	C	0.760					0.585
17	C	0.740					0.574
7	C	0.732					0.549
12	C	0.644					0.419
22	C	−0.644					0.437
26	C	0.587					0.375
29	C	−0.498					0.282
25	A	0.359		−0.358			0.272
6	E		0.831				0.750
16	E		0.764				0.669
1	E		−0.703				0.527
11	E		0.653				0.477
21	E		0.613				0.498
10	A			−0.761			0.630
5	A			−0.739			0.589
15	A			0.700			0.533
20	A			0.678			0.498
28	A			0.379			0.339
27	O				0.675		0.491
14	O				0.628		0.417
24	O				0.611		0.401
4	O				0.543		0.347
9	O				0.538		0.370
19	O				0.390		0.235
3	N					0.798	0.656
8	N					0.742	0.609
13	N					0.666	0.462
23	N					0.504	0.291
18	N					0.500	0.273
Proportion of variance (%)		11.9	9.9	8.5	8.2	8.1	
Cumulative proportion of variance (%)		11.9	21.8	30.4	38.6	46.7	

*Only moderate and significant factor loadings ≥ 0.30 are shown*. *O; openness to experience, C; conscientiousness, E; extraversion, A; agreeableness, N; neuroticism*.

### Validation of the Shortened 26-Item JBFS-SF

Three items (items 19, 25, and 28) had small or ambiguous loadings and were excluded. The following examines the 26-item JBFS-SF.

#### Structural Validity

**Figure 2:**
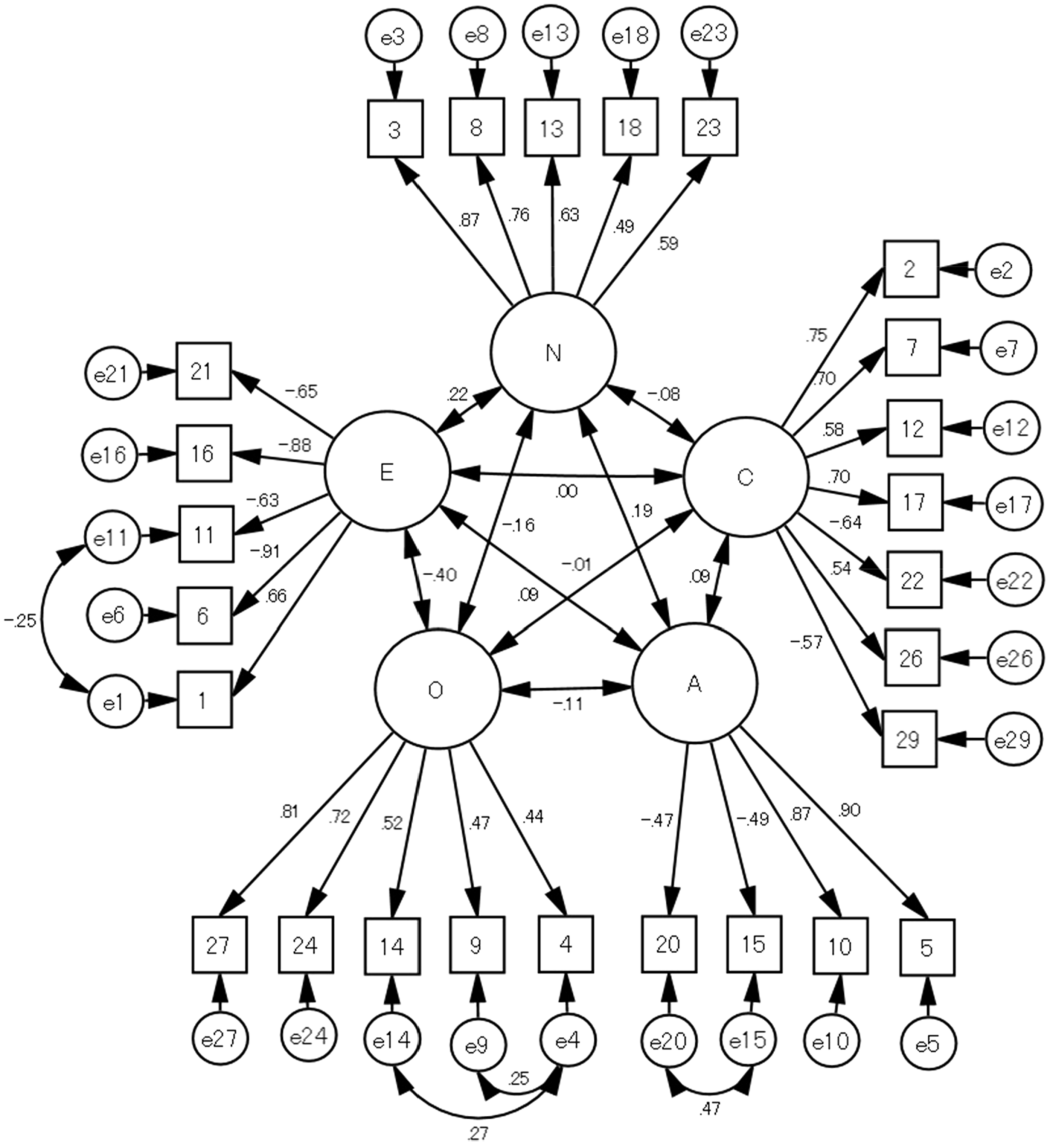
Confirmatory factor analysis of the five-factor structure of the shortened 26-item JBFS-SF (*N* = 781). O; openness, C; conscientiousness, E; extraversion, A; agreeableness, N; neuroticism.

Figure 2 indicates the results of the CFA assuming a covariant relationship between some error variables in Sample 2 (GFI = 0.907, AGFI = 0.886, CFI = 0.907, and RMSEA = 0.057). With multiple group structural equation modeling by sex, the result of CFA shows that the GFI = 0.875, AGFI = 0.862, CFI = 0.897, and RMSEA = 0.041. Table 3 shows the fit between four levels, assuming configural invariance, metric invariance, scalar invariance, or error variance invariance. Both ΔCFI and ΔTLI were ≤ 0.01 at all levels, indicating that there was no significant difference by sex.

**Table 3:** Measurement invariance tests across sex.

Model	*χ* ^2^	*df*	TLI	CFI	AIC	RMSEA	Δ*χ*^2^	Δ*df*	ΔTLI	ΔCFI
Configural invariance	1388.162	570	0.885	0.899	1771.113	0.051				
Metric invariance	1412.160	591	0.888	0.898	1765.565	0.052	23.998	21	0.003	−0.001
Scalar invariance	1434.149	606	0.890	0.898	1745.372	0.055	9.807	15	0.002	0.000
Error variance invariance	1494.072	636	0.891	0.894	1726.574	0.055	59.923	30	0.001	−0.004

*Df; degree of freedom, TLI; Tucker–Lewis index, CFI; comparative fit index, AIC; Akaike information criterion, RMSEA; root mean square error of approximation.*

#### Internal Consistency

The results of McDonald’s omega of each Big Five factor of the 26-item JBFS-SF were 0.74 for Openness to experience, 0.78 for Neuroticism, 0.82 for Agreeableness, 0.84 for Conscientiousness, and 0.85 for Extraversion.

#### Construct Validity

Table 4 shows the correlation coefficients for each Big Five factor of the 26-item JBFS-SF with depression, anxiety, autism spectrum, and social support. Within the Big Five factors, there was a weak positive correlation between Extraversion and Openness to experience. Neuroticism had a weak positive correlation with depressive symptoms and a moderate positive correlation with anxiety symptoms. The autistic trait subscale of the AQ-J-10 was not strongly correlated with any of the factors. As for social support, there was a weak positive correlation between the quantity of Social Support Questionnaire and Extraversion.

**Table 4:** Pearson’s Correlation between the shortened 26-item JBFS-SF, the PHQ-9, the GAD-7, the AQ-J-10, and the SSQ in Sample 1 (*N* = 845).

	O	C	E	A	N	PHQ9	GAD7	AQ-J-10	SSQ(n)	SSQ(q)
BFS-SF										
O	1									
C	0.06 (−0.01 to 0.12)	1								
E	0.38 (0.32 to 0.44)	−0.06 (−0.13 to 0.01)	1							
A	0.17 (0.10 to 0.23)	0.10 (0.03 to 0.16)	0.00 (−0.06 to 0.07)	1						
N	−0.16 (−0.23 to −0.09)	0.05 (−0.01 to 0.12)	−0.20 (−0.26 to −0.13)	−0.18 (−0.25 to −0.12)	1					
PHQ9	−0.07 (−0.13 to 0.00)	−0.15 (−0.22 to −0.09)	−0.07 (−0.14 to −0.00)	−0.10 (−0.17 to −0.04)	0.34 (0.28 to 0.40)	1				
GAD7	−0.01 (−0.08 to 0.05)	0.03 (−0.04 to 0.10)	−0.08 (−0.15 to −0.02)	−0.23 (−0.29 to −0.17)	0.50 (0.45 to 0.55)	0.56 (0.51 to 0.60)	1			
AQ-J-10	0.12 (0.05 to 0.18)	−0.11 (−0.17 to −0.04)	0.05 (−0.02 to 0.12)	−0.09 (−0.16 to −0.03)	0.04 (−0.02 to 0.11)	0.11 (0.04 to 0.17)	0.15 (0.08 to 0.22)	1		
SSQ(n)	0.09 (0.02 to 0.15)	0.09 (0.02 to 0.16)	0.32 (0.26 to 0.38)	0.12 (0.06 to 0.19)	−0.15 (−0.21 to −0.08)	−0.22 (−0.28 to −0.15)	−0.22 (−0.29 to −0.16)	−0.08 (−0.14 to −0.01)	1	
SQQ(q)	−0.02 (−0.09 to 0.04)	0.09 (0.02 to 0.15)	0.15 (0.08 to 0.21)	0.10 (0.02 to 0.16)	−0.07 (−0.14 to −0.00)	−0.19 (−0.25 to −0.12)	−0.17 (−0.24 to −0.11)	−0.04 (−0.10 to 0.30)	0.60 (0.56 to 0.64)	1

*BFS-SF: the shortened 26-item the Big Five Scale (O; openness, C; conscientiousness, E; extraversion, A; agreeableness, N; neuroticism), PHQ-9; Patient Health Questionnaire-9, GAD-7; Generalized Anxiety Disorder Screener-7, AQ-J-10; Autism Spectrum Quotient Japanese version, SSQ (n); Social Support Questionnaire—number, SSQ (q); Social Support Questionnaire—quality*.

## Discussion

This study examined the structure and validity of the JBFS-SF in a Japanese university student sample. We examined the 26-item version, removing 3 items with small or ambiguous loadings in the EFA. The results showed that this short version can act as a reliable and valid assessment of personality traits, simultaneously reducing the time and burden required to complete an assessment.

The results of the EFA showed that 29 items can be classified according to the expected five-factor structure, but three items had small loadings or moderate secondary loadings. Therefore, we dropped these three items and tested the reliability and validity of a 26-item version. In the present study, the CFA results showed that the 26-item JBFS-FS has adequate structural validity. Nevertheless, we could not compare the goodness-of-fit of the factor structure results with existing studies because a CFA has not previously been conducted.

Omega coefficients suggested good internal consistency. These results add to the evidence supporting the validity and reliability of the JBFS-FS. Furthermore, the results of multiple group structural equation modeling by sex indicated that the 26-item JBFS-SF had strict measurement invariance across genders.

In addition, we examined construct validity using associations between each Big Five Scale factor and potential outcomes of depression, anxiety, autism spectrum, and social support. First, we identified a positive correlation between Extraversion and Openness to experience of the Big Five factors, which was consistent with the results of the original Japanese version (Wada, 1996) and the short version (Namikawa et al., 2012). Second, we found a positive correlation between Neuroticism, the PHQ-9, and the GAD-7 scores. These relationships are consistent with previous findings showing that Neuroticism is strongly linked to depression and anxiety (Kotov et al., 2010; Hakulinen et al., 2015; Lyon et al., 2021). Third, the positive correlation between Extraversion and social support is consistent with a previous meta-analysis (Barańczuk, 2019). Third, while previous studies (Kanai et al., 2011; Strunz et al., 2015; Schwartzman et al., 2016) have commonly suggested positive correlations between Neuroticism and autistic traits, or negative correlations between Extraversion/Agreeableness and autistic traits, we did not find these features in the present study. Not observing such associations reported in the literature may be due to our sample being limited to basically healthy university students having relatively narrow variability in autistic traits. Previous studies used potentially sharper contrasts by comparing clinical samples with the healthy controls. Alternatively, this may be related to differences between the scales based on the Big Five Model. As noted in previous studies (Ohnogi, 2004; Namikawa et al., 2012), a comprehensive discussion on personality traits scales is required in the future.

Recently ultra-short scales measuring the Big Five personality traits have been reported in Japan (Oshio et al., 2012). How it compares with BFS-SF has not been examined.

### Limitations and Future Directions

The present study has some limitations of note. First, the participants in this study were university students below the threshold for clinical depression. Sample selection can have an impact on the results generated using some psychometric techniques, such as the EFA (Gaskin et al., 2017). Nevertheless, we were interested in the personality of university students. The sample was drawn from five different universities, both public and private in the second largest metropolitan area in Japan. We therefore argue that our sample would be fairly representative of university students in Japan. Results are not readily generalizable to younger adults in general, let alone the general population. Additionally, we did not compare the short form with the original BFS, NEO-FFI, or FFPQ. This limits the generalizability of our findings to only the short-form version.

### Conclusion

The 26-item JBFS-SF demonstrated adequate reliability and validity among Japanese university students when excluding three items with ambiguous loadings. From a practical point of view, this research adds evidence supporting the short form as a useful measure for assessing personality characteristics cross-culturally. Findings also showed that the Big five personality traits, as measured with this short form, may be used as a potential predictor and effect modifier of psychological symptoms in future research among university student populations.

## Data Availability Statement

The raw data supporting the conclusions of this article will be made available by the authors, without undue reservation.

## Ethics Statement

The Ethics Committee of Kyoto University School of Medicine have approved this study (Protocol # C1357). All participants provided written informed consent to participate in this study.

## Author Contributions

TF, SA, TIr, YS, TIw, TU, YL, and MSa designed this trial. RT, MSa, KY, YL, YN, HS, MSu, HI, TW, AT, TU, ES, and TF acquired the data. RT and MSa conducted the statistical analyses. RT and TF wrote the first draft of the manuscript. All authors contributed to the article and approved the submitted version.

## Funding

This research was funded by grants from Japan Agency for Medical Research and Development (AMED; grant number 20dk0307085), Japan Society for the Promotion of Science (JSPS; grant number 18K18643, 21K03049, 19F19110, and P19110), Suzuken Memorial Foundation, KDDI Foundation and Pfizer Health Research Foundation.

## Conflict of Interest

YS was employed by Goryokai Medical Corporation. TF reports grants and personal fees from Mitsubishi-Tanabe, personal fees from SONY, grants, and personal fees from Shionogi, COI outside the submitted work; in addition, TF has a patent 2020-548587 concerning smartphone CBT apps pending, and intellectual properties for Kokoro-app licensed to Mitsubishi-Tanabe. MSa reports personal fees from SONY outside the submitted work. AT has received lecture fees from Sumitomo Dainippon Pharma, Eisai, Janssen Pharmaceutical, Meiji-Seika Pharma, Mitsubishi Tanabe Pharma, Otsuka, and Takeda Pharmaceutical.

The remaining authors declare that the research was conducted in the absence of any commercial or financial relationships that could be construed as a potential conflict of interest.

## Publisher’s Note

All claims expressed in this article are solely those of the authors and do not necessarily represent those of their affiliated organizations, or those of the publisher, the editors and the reviewers. Any product that may be evaluated in this article, or claim that may be made by its manufacturer, is not guaranteed or endorsed by the publisher.
